# Endoscopic Stenting for Malignant Dysphagia in Patients with Esophageal Cancer

**DOI:** 10.3390/curroncol30070447

**Published:** 2023-06-21

**Authors:** Ryu Ishihara

**Affiliations:** Department of Gastrointestinal Oncology, Osaka International Cancer Institute, 3-1-69, Otemae, Chuo-ku, Osaka 541-8567, Japan; ryu1486@gmail.com; Tel.: +81-6-6945-1181; Fax: +81-6-6945-1902

**Keywords:** esophageal cancer, malignant dysphagia, radial force, stent, radiation, gastrostomy, adverse event

## Abstract

Malignant dysphagia is a common problem in patients with esophageal cancer. Endoscopic stenting can resolve dysphagia caused by malignant stricture; however, controversy exists regarding the use of esophageal stenting for the treatment of malignant stricture, including whether stenting or radiotherapy is superior, whether stenting before or after radiotherapy is safe, whether stenting before or after chemotherapy is safe, and whether low-radial-force stents are safer than conventional stents. Among treatment options for malignant dysphagia, stenting may have some disadvantages in terms of pain relief and the risk of adverse events compared with radiotherapy and in terms of survival compared with gastrostomy. Additionally, the risk of stent-related adverse events is significantly associated with prior radiotherapy. The risk of perforation is especially high when a radiation dose of >40 Gy is delivered to the esophagus after stenting, whereas perforation is not associated with prior chemotherapy or additional chemotherapy after stenting. Nevertheless, stenting remains an important palliative option, especially for patients with a short life expectancy and a strong desire for oral intake, because stenting can facilitate a more rapid improvement in dysphagia than radiotherapy or gastrostomy. The application of a low-radial-force stent should be considered to reduce the risk of adverse events, especially in patients with prior radiotherapy.

## 1. Introduction

Esophageal cancer is the sixth most common cause of cancer-related mortality, with 455,800 new cases and 400,200 deaths worldwide in 2012 [1]. This cancer has a poor prognosis with an overall 5-year survival rate of <20% [2]. The two pathological types of esophageal cancer are esophageal adenocarcinoma and esophageal squamous cell carcinoma. Esophageal squamous cell carcinoma is the predominant type of esophageal cancer in South America, Africa, and Asia. Conversely, esophageal adenocarcinoma is increasing in frequency and is the predominant type of esophageal cancer in the United States and Europe [1]. Various treatments, including surgery, chemotherapy, radiotherapy (RT), and endoscopic therapy, are indicated as curative treatments for esophageal cancer. Although esophagectomy has been the mainstay of curative treatment for esophageal cancer, substantial proportions of patients are unsuitable for esophagectomy at presentation because of tumor growth into the lungs, bronchi, or aorta; extensive lymph node metastases; or distant metastases to the liver or lungs. In addition, severe comorbidities or a poor performance status prevents treatment by esophagectomy. Chemoradiotherapy (CRT) is another curative treatment option for esophageal cancer; however, the outcome of this treatment for patients with inoperable cancer is unfavorable. Thus, the treatment outcome of esophageal cancer remains poor [2], highlighting the importance of care for patients with incurable cancers.

Most patients with incurable cancers will require various palliative treatments. These patients frequently present with cancer-related complications that increase in number and severity as the disease advances, causing pain, malnutrition, a deteriorated performance status, and a deterioration in the quality of life (QoL) [1]. Dysphagia secondary to local tumor extension is one of the most frequent symptoms among patients with incurable and advanced esophageal cancer [3].

Multiple palliative treatment modalities are available for patients with dysphagia due to local tumor extension, including a bypass operation, gastrostomy, palliative RT, and esophageal stenting [4]. The optimal knowledge and use of the various treatment approaches for patients with unresectable and metastatic esophageal cancer will contribute to achieving the best possible outcomes in individual patients. Patients with incurable esophageal cancer require palliative treatment, which should be the safest, most effective treatment available and should also be cost-effective. In addition, the palliative treatment that is selected should be based on the location and physical features of the tumor, the age and general condition of the patient, the tumor spread, and the predicted survival interval. Endoscopic stenting is characterized by a prompt improvement in dysphagia and is an important treatment option for this condition. However, controversy exists regarding the use of esophageal stenting for the treatment of malignant stricture, including whether stenting or RT is superior, whether stenting before or after RT is safe, whether stenting before or after chemotherapy is safe, and whether low-radial-force stents are safer than conventional stents. This article presents a review of the literature based on recently published Japanese guidelines for esophageal cancer [5,6,7].

## 2. Selection of Articles

The reviewed articles [8,9,10,11,12,13,14,15,16,17,18,19,20,21,22,23,24,25,26,27,28] were selected from among those extracted through a systematic review of the guidelines [5,6,7] to avoid bias from the article selection process. However, 14 articles [29,30,31,32,33,34,35,36,37,38,39,40,41,42] were added for the further exploration of topics that were not included in the guidelines. In the guidelines, the Japan Medical Library Association was entrusted with a systematic search of the literature published from January 2000 to August 2020. The PubMed database, Cochrane Library database, and ICHUSHI-Web database were used to search for articles. Moreover, some articles that had escaped retrieval by the systematic search were added through a manual search by the guideline committee members.

## 3. Stenting and Radiation for Palliation of Malignant Dysphagia

RT and CRT for dysphagia relief in the palliative setting were compared in a randomized trial including 220 patients [8]. This study showed no difference in the median survival time between the CRT group (6.9 months) and RT group (6.7 months). CRT showed a slightly better improvement in dysphagia than RT (45% vs. 35%, respectively; *p* = 0.13); however, the difference was not statistically significant. The incidence of grade 3 and 4 adverse events was significantly higher in the CRT group than in the RT group (36% vs. 16%, respectively; *p* = 0.0017). Palliative CRT showed a modest, but not statistically significant, increase in dysphagia relief compared with RT, with a cost of increased toxicity. This study concluded that RT alone should be considered a safe and well-tolerated treatment for malignant dysphagia in the palliative setting. Because CRT is considered to be superior to RT in many situations, further studies are required to confirm the efficacy of CRT for the palliation of malignant dysphagia.

Palliative RT and esophageal stenting are the most frequently used therapies for dysphagia due to incurable esophageal cancer, and their relative effectiveness was evaluated in two studies [9,10]. Brachytherapy and stenting were compared in a randomized study including 209 patients [9]. The median survival was not significantly different between the two groups (*p* = 0.23). The dysphagia score improved more rapidly after stent placement than after brachytherapy. However, at 30 days after treatment, the improvement in the dysphagia score no longer differed significantly between the stenting and brachytherapy groups. After 30 days, the dysphagia score was better after brachytherapy than after stenting, although this difference became smaller after about 350 days. Brachytherapy had fewer complications than stenting (5% vs. 13%, respectively), which was mainly due to a decreased incidence of late hemorrhage. Brachytherapy also had better health-related QoL scores than brachytherapy.

External RT and stenting were compared in a retrospective study including 1957 patients [10]. In a multivariable analysis of the 527 patients for whom preintervention and postintervention dysphagia scores were available, stenting showed a larger, more rapid improvement in dysphagia than RT over time (*p* = 0.019). However, compared with esophageal stent placement, palliative RT was associated with more rapid and persistent pain relief over time (*p* = 0.0010) and a decreased risk of adverse effects (21.7% in stenting group vs. 12.4% in RT group; *p* = 0.001).

These studies show that palliative RT is associated with a lower risk of adverse events and improved pain control. Based on these studies, palliative RT is recommended for the treatment of patients with cStage IVB esophageal cancer presenting with obstruction in the recently published Japanese guidelines. Notably, however, stenting should be offered to patients with a short life expectancy because of its rapid efficacy for dysphagia.

## 4. Stenting and Gastrostomy for Palliation of Malignant Dysphagia

In patients who have a history of RT or cannot tolerate RT, stenting and gastrostomy are the major treatment options. The outcomes of these two procedures were compared in a retrospective study including 568 patients [11]. In this study, the main analyses were conducted in 188 patients using propensity score matching. Although there was no significant difference in the body weight change or occurrence of procedure-related adverse events between the two groups, the gastrostomy group showed slightly better survival after adjustment by multivariate analysis (hazard ratio, 0.69; 95% confidence interval (CI), 0.50–0.95). The advantages of stenting are the rapid relief of dysphagia and satisfaction derived from the ability to eat, whereas the disadvantages are adverse events such as chest pain, migration, mediastinitis, perforation, and bleeding. The advantages of gastrostomy are a stable nutritional status and the safety of the procedure, whereas the disadvantages are dissatisfaction with the inability to eat and esophageal-obstruction-related symptoms such as the reflux of saliva. These treatment characteristics should be taken into consideration when a strategy is selected. The author’s recommendation depends largely on the patient’s preference; stenting is recommended for those who wish to eat despite the risks of stenting.

## 5. Influence of RT and Chemotherapy before Stenting

RT and CRT are major treatment options for patients with esophageal cancer, especially for patients with cStage IVB esophageal cancer presenting with malignant dysphagia. The influence of prior RT on the efficacy and adverse events of stenting was evaluated in a systematic review for esophageal cancer practice guidelines [7]. In the systematic review, seven observational studies were selected for a qualitative systematic review [12,13,14,15,16,17,18]. Only one study evaluated the efficacy of stenting in terms of dysphagia and found no significant difference between patients with and without prior RT. The systematic review of these seven studies (Table 1) showed that the risk of adverse events, such as bleeding, fistula, and perforation, increases with the history of RT. The risk difference was 0.14 (95% CI, 0.04–0.24) between patients with and without prior RT.

Although prior RT is a risk factor for adverse events of stenting, the incidence of adverse events in the two most recent studies [17,18] was lower than that in prior studies. These two studies [17,18] suggested that the use of a stent with a low radial force may help to reduce the risk of adverse events.

The influence of prior chemotherapy without radiation on the efficacy and adverse events of stenting was evaluated in a systematic review for esophageal cancer practice guidelines [7], and four observational studies were selected for evaluation [15,16,19,20]. In one of the studies [19], the patient survival, stent patency, and improvement in dysphagia were evaluated according to the presence or absence of prior chemotherapy. The median duration of stent patency was significantly shorter in patients with than without prior chemotherapy, but it was still relatively long at 162 days. A systematic review of four studies (Table 2) focusing on adverse events, such as bleeding, fistula, and perforation, showed no significant difference between patients with and without prior chemotherapy. However, in one study [20], stent migration was more common in patients with than without prior chemotherapy (12.5% vs. 4.8%, respectively).

## 6. Influence of RT and Chemotherapy after Stenting

Stent therapy has been applied to patients with a short life expectancy because it can achieve an early improvement in malignant dysphagia. Subsequent additional therapy such as RT and chemotherapy can be conducted if patients can tolerate such treatment. The validity of additional RT after stenting was evaluated in a systematic review for esophageal cancer practice guidelines [7]. In the systematic review, two randomized studies [21,22] were extracted for the evaluation of efficacy and adverse events, and five observational studies [23,24,25,26,27] were extracted for the evaluation of adverse events of additional RT after stenting.

A randomized study that mainly included patients with squamous cell carcinoma (83.5% (66/79) of patients) showed a significant improvement in the duration of dysphagia relief and overall survival in the RT group compared with the non-RT group [21]. Another randomized study that mainly included patients with adenocarcinoma (64.8% (129/199) of patients) showed no difference in survival or recurrent dysphagia at 12 weeks between the RT group and the non-RT group. A meta-analysis of these two randomized studies showed that the risk difference (95% CI) was 0.11 (−0.18 to 0.39), which slightly favored the RT group, although the difference was not statistically significant.

The risk of adverse events associated with additional RT after stenting was evaluated in seven studies, including two randomized studies [21,22] and five observational studies [23,24,25,26,27]. In two studies [21,25], the risk of adverse events was not different between the RT group and the non-RT group, although the frequency of adverse events was not described. The risks of adverse events in the other five studies are shown in Table 3. In these studies, the risk of perforation was especially high when a radiation dose of >40 Gy was delivered to the esophagus after stenting. This finding suggests that additional RT may benefit patients with malignant dysphagia, but additional RT with a dose of >40 Gy should be avoided in view of the risk of adverse events.

In the systematic review for the evaluation of additional chemotherapy after stenting, two observational studies [24,28] were extracted. Neither study showed a significant improvement in the stent patency. One of the studies showed better survival after stenting in the chemotherapy group than in the non-chemotherapy group [24]. However, considering that this was a retrospective observational study, the survival analysis may have been biased by the patient selection process because patients’ conditions are usually better in the chemotherapy group than in the non-chemotherapy group.

In the evaluation of adverse events, neither study showed a significant difference between the chemotherapy group and non-chemotherapy group. However, stent migration was significantly more frequent in the chemotherapy group than in the non-chemotherapy group. Considering that stent migration does not usually have serious consequences [24,28], there is no reason to avoid chemotherapy after stenting.

## 7. Stent Type and Efficacy

Recurrent obstructions are caused by tumor ingrowth or overgrowth, stent migration, or food impaction in the stent. Partially covering the stent with a membrane has been shown to be superior to using uncovered stents, with a reduced rate of tumor ingrowth through the mesh of the stent [29]. However, the proximal and distal flares of a partially covered stent are not covered and still have the potential for ingrowth. Fully covered stents may overcome the limitation of tumor ingrowth, but the risk of migration may be increased.

During the last decade, three randomized trials were conducted to compare the effectiveness and risk of different types of stents [30,31,32], and two of these studies evaluated the effectiveness of fully covered stents [30,31]. Didden et al. [30] compared recurrent obstruction, adverse events, and health-related QoL between fully covered and partially covered stents in 98 patients. Recurrent obstruction after stent placement was similar between the two types of stents: 19% for fully covered stents and 22% for partially covered stents (*p* = 0.65). The frequency of adverse events was also similar between the two groups, with major adverse events occurring in 38% and 47% of patients with fully covered and partially covered stents, respectively (*p* = 0.34). No significant differences were seen in health-related QoL or migration. Stent removal was required in 4 of 97 patients. The reasons for self-expandable metallic stent removal were intolerable pain (*n* = 2), symptomatic tracheal compression (*n* = 1), and insufficient symptom relief (*n* = 1).

Persson et al. [31] compared fully covered stents with partially covered stents in terms of stent migration in 95 patients. Stent migration during the total study period occurred in 37.2% of patients in the partially covered stent group but in only 20.0% of patients in the fully covered stent group. There were no significant differences in either the stent migration distance or the migration frequency. There was a tendency toward the achievement of better dysphagia relief in the fully covered stent group than in the partially covered stent group, but the difference was not statistically significant (*p* = 0.081). Fully covered stents failed to show a benefit in terms of dysphagia relief, but these stents were not associated with increased adverse events or migration. Thus, fully covered stents remain an optional treatment for malignant dysphagia.

A stent may lead to acid reflux when it is placed across the gastroesophageal junction. A stent with an anti-reflux valve was designed to prevent acid reflux through the stent. The third of the three above-mentioned randomized studies [32] evaluated the efficacy and safety of this stent in 60 patients. The dysphagia scores, gastroesophageal reflux disease (GERD) symptom scores, and frequency of aspiration pneumonia were not different between stents with and without an anti-reflux valve during the follow-up period. The GERD symptom scores were similar between the two stents, implying either that the valve was not effective or that proton pump inhibitor therapy could have masked the symptoms of GERD.

In addition to the stent covering and the anti-reflux valve, the axial and radial forces of the stent are major determinants of the stent’s properties [33]. The radial force is the force that stents exert as they resist compression by the pressure of the esophageal wall; it is also the force that stents exert on the lumen as they expand to their original nominal diameter. The axial force is the force exerted on the luminal wall when the stent is in a curved position. The radial and axial forces of various stents were evaluated using an in vitro testing model [33].

Pressure on the esophageal wall is mainly determined by the stent radial force. A higher radial force may better stabilize the stent position; however, the strong compression may also cause adverse events such as perforation and bleeding. Two recent studies from Japan showed a reduced risk of adverse events in patients treated with low-radial-force stents [17,18]. Iwagami et al. [18] compared 51 procedures performed with low-radial-force stents and 56 procedures performed with high-radial-force stents. Severe adverse events occurred more frequently in procedures performed with high- than low-radial-force stents (14% (8/56) vs. 2% (1/51), respectively; p = 0.03). In patients who had undergone prior RT, severe adverse events were also more frequent in procedures performed with high- than low-radial-force stents (36% (4/11) vs. 0% (0/13), respectively; *p* = 0.03). There was no significant difference in re-obstruction or migration between procedures performed with low- and high-radial-force stents (*p* = 0.59 and *p* = 1, respectively). A low-radial-force stent may reduce the risk of severe adverse events after stenting without compromising its efficacy, which may be a preferred option for patients with malignant dysphagia, especially after RT.

While stents provide immediate and efficient palliation of dysphagia, the recurrence of dysphagia after stenting leads to a worsening QoL and requires further intervention, such as re-stenting. Although RT takes a longer time to relieve the symptoms of dysphagia caused by esophageal cancer, it usually allows for oral intake and is associated with fewer treatment-related adverse events. The combination of stents and radiation appears to be a viable and effective palliative therapy for patients with malignant dysphagia. However, there is concern regarding an increased incidence of adverse events. Brachytherapy delivers radiation precisely inside the esophageal cancer tissue, thus sparing surrounding normal organs, such as the aorta and lungs. The placement of ^125^I brachytherapy seeds on the esophageal stent can alleviate dysphagia and prevent tumor extension into the stent lumen. Stents loaded with ^125^I seeds have been developed to combine the immediate relief of dysphagia by stenting with the long-term effects of radiation [34,35]. In such stents, an ^125^I radioactive seed is preloaded in the sheath and attached to the outer surface of the stent prior to stent insertion.

In a single-center randomized controlled trial [34], 27 patients in the irradiation stent group and 26 patients in the stent-alone group showed significantly better dysphagia grades in the first month after stenting than before stenting. However, the irradiation stent group showed better dysphagia grades than the stent-alone group after 2 months. The median and mean survival times were significantly better in the irradiation stent group than in the stent-alone group (*p* < 0.001). This study suggests that esophageal stents loaded with ^125^I seeds offer advantages over conventional coated stents in terms of a reduced duration of dysphagia and prolonged survival. A multicenter randomized controlled trial [35] was conducted to confirm the efficacy of stents loaded with ^125^I seeds. In that study, 160 patients were randomly allocated to receive either a stent loaded with ^125^I seeds or a conventional stent. The median overall survival was better in the irradiation stent group than in the stent-alone group (177 vs. 147 days, respectively; *p* = 0.0046). The mean dysphagia score in the irradiation stent group remained significantly better than that in the stent-alone group from 1 month after stent insertion until the final follow-up. The main treatment-related complication was severe chest pain in 23% of patients in the irradiation stent group and 20% of patients in the stent-alone group. This study showed that stents coated with ^12^⁵I seeds provided better dysphagia relief and longer survival than conventional covered stents without increasing the incidence of adverse events in patients with malignant dysphagia. A recent meta-analysis of four randomized controlled trials and four retrospective studies compared the efficacy of irradiation stents and conventional stents [36]. The two groups had comparable dysphagia scores (*p* = 0.80), as well as comparable rates of stent restenosis (odds ratio (OR) = 0.97, *p* = 0.89), stent migration (OR = 0.81, *p* = 0.63), severe chest pain (OR = 1.05, *p* = 0.81), bleeding (OR = 1.53, *p* = 0.16), aspiration pneumonia (OR = 0.72, *p* = 0.38), and fistula formation (OR = 1.47, *p* = 0.44). The pooled times to restenosis and survival were both significantly longer in the irradiation stent group than in the stent-alone group (*p* = 0.04 and *p* < 0.0001, respectively).

The incorporation of drugs and stents has resulted in a new type of stent called a drug-eluting stent [37,38]. Drug-eluting stents allow for the sustained release of therapeutic agents while simultaneously providing the stent function. Controlled drug elution is often achieved using a drug-impregnated polymer membrane or coating that acts as a drug reservoir for sustained drug release [39]. Several approaches and drug–polymer combinations have been employed to create drug-eluting stents that deliver chemotherapy drugs, including 5-fluorouracil, paclitaxel, and docetaxel, for the treatment of esophageal cancer [40,41].

A paclitaxel or 5-fluorouracil stent was prepared by covering a nitinol stent with a bilayer polymer film consisting of a layer of 50% paclitaxel or 5-fluorouracil and a layer of a drug-free backing [42]. The in vivo evaluation of their performance in a porcine model revealed localized drug accumulation in the esophageal lumen relative to other organs, although stent migration was noted as a limitation. Although the drug-eluting stent is a promising device to realize rapid dysphagia relief, with long-term dysphagia relief provided by chemotherapy, clinical studies are needed to confirm its efficacy.

## 8. Other Topics

Systemic chemotherapy is usually recommended for patients with cStage IVB esophageal cancer. Previous studies have shown that systemic therapy results in dysphagia improvement in 72% to 90% of patients 2 to 6 weeks after the initiation of therapy. The dysphagia relief can last from a few weeks to a few months or longer if the patient continues to respond to the therapy [43,44]. A Cochrane review of systemic therapy for esophageal cancer concluded that systemic therapy improves dysphagia; conversely, the authors recommended against using chemotherapy alone for dysphagia palliation in patients with esophageal cancer because of the high incidence of recurrent symptoms [45]. However, chemotherapy is evolving with the addition of immunotherapy, and the treatment outcomes of dysphagia palliation are expected to improve [46,47,48].

There are many options for the treatment of malignant dysphagia in patients with esophageal cancer. The development of a proper treatment strategy is important. For example, a chemotherapy-naïve patient with widespread metastatic disease may benefit more from chemotherapy. By contrast, a patient who has experienced disease progression on two or more lines of chemotherapy but has small-volume metastatic disease could benefit more from stenting or RT.

## 9. Conclusions

Stenting is a treatment option for patients with esophageal-cancer-induced dysphagia, but it may have some disadvantages in terms of pain relief and the risk of adverse events compared with RT and in terms of survival compared with gastrostomy. In addition, the risk of stent-related adverse events is associated with prior or additional RT.

However, stenting remains an important palliative option, especially in patients who have a short life expectancy and strong desire for oral intake, because stenting can facilitate a rapid improvement in dysphagia. The application of a low-radial-force stent should be considered to reduce the risk of adverse events, especially in patients with prior RT.

Based on the evidence gathered to date and the author’s personal experience, the following three strategies are recommended. First, for patients with a good general condition and mild dysphagia (able to eat a liquid diet or soft diet), systemic chemotherapy is recommended. Second, for patients with a good general condition and severe dysphagia (unable to eat a liquid diet), CRT or RT is recommended. Although a previous study [8] failed to show the superiority of CRT for dysphagia relief, the author usually prefers CRT for patients who can tolerate it or patients who have extensive metastasis because the efficacy of CRT is usually higher than that of RT. Third, for patients with a poor general condition, the author recommends stenting because it can achieve a rapid improvement in dysphagia.

## 10. Future Perspectives

Stenting is a promising treatment option for malignant dysphasia; however, its relative efficacy compared with other treatment options, such as RT or gastrostomy, has only been investigated in retrospective studies [10,11]. These retrospective studies may have been biased by the patient selection process and may have shown poorer outcomes in the stent group because stenting is usually indicated for patients with a poor general condition. A randomized study or prospective study with propensity score matching is required to accurately evaluate the efficacy of stenting with other treatment options.

Another important issue is the evaluation of stents with additional functions, such as stents loaded with radioactive ^125^I seeds [34,35]. Although these specialized functions may enrich the efficacy of the stents, the use of such stents is limited in some areas. A comparison of these specialized stents with community-standard treatment in other areas of the world would elucidate their real value.

## Figures and Tables

**Table 1:** Risk of adverse events in patients with and without prior RT.

First Author Year	Adverse Events	
	Prior RT	No Prior RT
Song HY 2002 [12]	8.5% (4/47 pts)	2.1% (1/48 pts)
	Fistula 2 pts Severe bleeding 2 pts	Fistula 1 pt
Lecleire S 2006 [13]	26.8% (15/56 pts)	6.7% (4/60 pts)
	Perforation or fistula 9 pts Severe bleeding 6 pts	Fistula 1 pt Severe bleeding 2 pts
Park JY 2012 [14]	11.3% (8/71 pts) *^1^	2.7% (2/74 pts) *^1^
	Fistula 5 Airway narrowing 2 Both of above 1	Fistula 1 Airway narrowing 1
Qiu G 2013 [15]	50.9% (29/57 pts)	14.3% (5/35 pts)
	Fistula 5 Bleeding 24	Fistula 2 Bleeding 3
Liu SY 2016 [16]	29.3% (41/140 pts) *^2^	3.4% (13/379 pts) *^2^
	Massive bleeding 41 pts	Massive bleeding 13 pts
Uesato M 2017 [17]	1.9% (1/52 pts)	5.6% (2/36 pts)
	Perforation 1 pt	Perforation 1 pt Bleeding 1 pt
Iwagami H 2021 [18]	16.7% (4/24 pts)	6.0% (5/83 pts)
	Bleeding 3 pts Perforation 3 pts Severe pain 1 pt	

Adverse events mainly include bleeding, fistula, and perforation. RT: radiotherapy; pts: patients. *^1^ Only airway complications were evaluated. *^2^ Only massive bleeding was evaluated.

**Table 2:** Risk of adverse events in patients with and without prior CT.

First Author Year	Prior CT	No Prior CT	Analysis of Adverse Events
Qiu G 2013 [15]	32 pts	60 pts	No association between fatal bleeding/pneumonia and prior CT.
	Fatal bleeding 7 pts	Fatal bleeding 11 pts	
Liu SY 2016 [16]	334 pts	185 pts	No association between massive bleeding and prior CT.
	Massive bleeding 30 pts	Massive bleeding 24 pts	
Iwasaki H 2017 [19]	24 pts	29 pts	No association between major adverse events *^1^ and prior CT.
	Bleeding 3 pts Perforation 4 pts Fever 6 pts Aspiration pneumonia 3 pts		
Bakheet N 2020 [20]	64 pts	41 pts	No association between adverse events and prior CT.
	Bleeding 1 pt	Fistula 1 pt	

CT: chemotherapy; pts: patients. *^1^ Major adverse events include bleeding, perforation, fistula, aspiration pneumonia, and fever.

**Table 3:** Risk of adverse events in patients who received additional RT.

First Author Year	RT Dose/Fr	Adverse Events
Adamson, D., 2021 [22]	20 Gy/5 Fr or 30 Gy/10 Fr	Perforation (1%), bleeding (2%)
Jiang, X.J., 2012 [23]	CRT 45 Gy/25 Fr	Perforation (24%)
Park, J.H., 2012 [24]	Median 48 Gy (including CRT)	Perforation (21%)
Lu, Y.F., 2018 [26]	CRT median 47.5 Gy	Perforation (87.5%)
Rueth, N., 2012 [27]	Median 50.4 Gy	Perforation (11%)

RT: radiotherapy; Fr: fraction; CRT: chemoradiotherapy.

## Data Availability

Not applicable.
